# Supplementary material for: Coordinated cortical thickness alterations across six neurodevelopmental and psychiatric disorders
Source: Nat Commun. 2022 Nov 11;13:6851. doi: 10.1038/s41467-022-34367-6 (PMC9652311; doi:10.1038/s41467-022-34367-6)
Supplement: Supplementary file 1 — Supplementary Information [file 41467_2022_34367_MOESM1_ESM.pdf]

Coordinated cortical thickness alterations across six neurodevelopmental and psychiatric disorders

Supplementary Information

MD Hettwer*, S Larivière, BY Park, OA van den Heuvel, L Schmaal, OA Andreassen, CRK Ching, M Hoogman, J Buitelaar, D van Rooij, DJ Veltman, DJ Stein, B Franke, TGM van Erp, ENIGMA ADHD Working Group, ENIGMA Autism Working Group, ENIGMA Bipolar Disorder Working Group, ENIGMA Major Depression Working Group, ENIGMA OCD Working Group, ENIGMA Schizophrenia Working Group, N Jahanshad, PM Thompson, SI Thomopoulos, RAI Bethlehem, BC Bernhardt, SB Eickhoff, SL Valk*

* Correspondence to Meike D. Hettwer (meike.hettwer@maxplanckschools.de) and Sofie L. Valk (valk@cbs.mpg.de)

Supplementary Methods

Population connectivity data

Population connectivity data was derived from a healthy young adult sample (n=207; 83 males, mean age±SD=28.73±3.73 years, range=22-36 years) from the Human Connectome Project (HCP[1]). Resting-state functional data underwent distortion and motion corrections, intensity inhomogeneity corrections and intensity normalization, brain extraction, normalization to MNI152 space and projection onto the cortical surface. Pre-processing of diffusion MRI data included b0 intensity normalization as well as corrections for head motion, susceptibility distortion and eddy currents. Both functional and structural connectivity data was parcellated according to the Desikan-Killiany atlas[2].

Subject-level functional connectivity matrices were generated by pair-wise correlations between time series of 68 cortical parcels and 12 sub-cortical structures. Z-scored subject-level data was accumulated to derive a group-average functional connectome[3]. Structural connectivity matrices included in the ENIGMA Toolbox[1] are based on anatomically constrained tractography, where reconstructed streamlines were generated for 68 parcels and 12 sub-cortical structures. Using distance-dependent thresholding, a group-average structural connectome was derived and log-transformed.

Spin tests

Wherever possible, we implemented spin tests as included in the ENIGMA Toolbox to assess the significance of spatial similarities via permutations. Spatial permutation tests correct for auto-correlations between smooth spatial maps by generating null models of respective spatial overlaps. That is, coordinates of cortical data are inflated to a sphere and rotated 1000 times, matching phenotypic data to different parcels in every permutation[1,2]. This step is repeated for both maps. Significance is determined by testing initial correlation coefficients against the null distributions retrieved by correlating rotated spatial maps.

Supplementary Discussion

Association between co-alteration hubs, shared susceptibility, and epicenters

For a better understanding of what is reflected in transdiagnostic co-alteration hubs (**Figure S1A**), we further examined their association with the topography of shared susceptibility and cortical thickness increases compared to decreases. First, we took the absolute Cohen's *d* maps for each disorder and rescaled each map between 0-1 to get the spatial patterns of illness effects independent from offsets in average effect between disorders. We then took the mean of the six resulting maps to receive a "hit map", where regions with values close to 6 (due to the number of included disorders) are most strongly and consistently affected across disorders (**Figure S1B**). Second, we thresholded each Cohen's *d* map to get the top 20% percent of regions showing thickness decreases or increases, respectively. We then binarized and summed these maps to observe which regions most strongly and consistently show thickness increases or reductions across disorders (**Figure S1C&D**). Last, we computed potential disease epicenters as described in the Main text using the "hit map" instead of co-alteration hubs to examine to which degree epicenters indeed relate to shared impact rather than coordinated impact (**Figure S1E&F**).

Robustness of cross-disorder co-alteration network hubs

In order to assess stability of cross-disorder co-alteration hub maps, we recreated hub maps based on cross-disorder inter-regional correlation matrices thresholded at 90%, 70% and 50%. All three alternative hub maps correlated significantly with original co-alteration hubs (90% threshold: r = 0.78; 70% threshold: r = 0.91; 50% threshold: r = 0.62; all p_{spin} < .0001). Correcting the cross-disorder co-alteration matrix for sample sizes via a partial correlation did not impact resulting hubs (correlation of *n*-corrected co-alteration hub map with original co-alteration hub map: r = 0.89, p_{spin} < .0001; **Figure S2A**).

Robustness of transdiagnostic gradients

To assess robustness of the first two transdiagnostic gradients, we compared them to gradients derived from manipulating analysis steps in the gradient computation and changing parameters in the BrainSpace Toolbox[3] (see **Figure S2B**). First, since there were generalized differences in the strength of disease impact on cortical thickness across disorders, we mean-corrected the initial correlation matrix by using a partial correlation coefficient. Gradients computed based on this mean-corrected matrix correlated highly with original gradients (G1: r = 0.94; G2: r = 0.87; both p_{spin} < .0001). Second, original gradients correlated highly with gradients derived using a different non-linear dimension reduction method (Laplacian eigenmap: G1: r = 1; G2: r = 0.99; both p_{spin} < .0001) or a linear dimension reduction method (principal component analysis: G1: r = 1; G2: r = 0.99; both p_{spin} < .0001). Third, even though data was normally distributed, it yielded relatively sparse data points for each inter-regional correlation. We therefore tested whether the use of Spearman's rho instead of Pearson's r in computing the initial correlation matrix influences gradient organization. Gradients based on Spearman's rho

correlation coefficients correlated highly with the original gradients (Spearman: G1: $r = 0.94$; G2; $r = 0.84$). Fourth, gradients were not impacted by correcting for sample sizes of underlying disorder samples as tested by including sample sizes as covariates in the computation of the cross-disorder co-alteration matrix via partial correlation (G1: r = 0.99; G2: r = 0.96; both $p_{spin} < .0001$). Last, original gradients also correlated highly with gradients for which cut-off values (i.e. sparsity) of the correlation matrix was manipulated (sparsity of 90%: G1: $r = 0.97$; G2: $r = 0.92$; sparsity of 70%: G1: $r = 0.99$; G2: $r = 0.96$; sparsity of 50%: G1: $r = 0.94$; G2: $r = 0.80$; all $p_{spin} < .05$). Overall, original gradients were robust against multiple parameter manipulations.

Influence of individual disorders on gradient organization

To assess whether gradient organization was differentially impacted by individual disorders, we performed leave-one-disorder-out analyses and correlated resulting gradients with original gradients G1 and G2 (**Figure S2B**). We observed that gradients were generally robust against leaving out single disorders (w/o ADHD: $r_{G1} = 0.95$; $r_{G2} = 0.77$; w/o BD: $r_{G1} = 0.99$; $r_{G2} = 0.86$; w/o SCZ: $r_{G1} = 0.98$; $r_{G2} = 0.94$; w/o OCD: $r_{G1} = 0.99$; $r_{G2} = 0.83$; w/o MDD: $r_{G1} = 0.99$; $r_{G2} = 0.97$; all $p_{spin} < .0001$). However, this was not the case for ASD (without ASD: $r_{G1} = 0.10$; $r_{G2} = 0.01$). Leaving out ASD in gradient computations appeared to lead to a switch in features to be reflected in principal and secondary gradients, as was observed in significant correlations of the principal gradient without ASD with the original G2 ($r = 0.86$, $p_{spin} < .001$) and of the secondary gradient without ASD with the original G2 ($r = 0.48$, $p_{spin} < 0.01$). This finding is not surprising, as cortical thickness alterations in ASD show a spatial pattern that is highly similar to G1 and thus likely strengthens the weight of features then represented in G1.

The third to eighth gradients of transdiagnostic co-alteration in cortical thickness

We additionally studied the third to eighth transdiagnostic gradients which are depicted in **Figure S3**. The third gradient traversed from sensory-limbic to lateral temporal cortex, while the fourth segregated (para)limbic from lateral prefrontal regions. The fifth gradient had a bilateral axis in orbitofrontal and limbic cortex on the one hand, and superior parietal cortex on the other hand. The sixth gradient was characterized by hemispheric asymmetry, distinguishing entorhinal and superior temporal sulcus from the temporoparietal junction and paracentral lobule in the left hemisphere, but lingual gyrus and cuneus from paracentral lobule and pars triangularis in the right hemisphere. Lastly, the seventh gradient captured a segregation between sensory-limbic and heteromodal cortices.

G1 captures segregation of functional disease epicenters

As we noticed that frontal and temporal disease epicenters appear to be segregated by G1 (**see Figure S4**) suggesting differential impact on co-alteration network organization, we performed a follow up analysis to confirm this assumption. In order to evaluate whether whole-brain cross-disorder disease impact shows different covariance patterns for frontal and temporal epicenters, we i) extracted cross-disorder inter-regional correlations from the 68 x 68 correlation matrix

(see **Figure 1C & 2A** of the main manuscript) for all frontal and temporal disease epicenters, respectively, and computed their degree centrality as the sum of all correlations of each epicenter parcel. ii) We extracted cross-disorder whole-brain structural covariance for two representative epicenters, the left *pars orbitalis* and entorhinal cortex, which emerged as the two strongest (functional) epicenters. Both approaches revealed that frontal epicenters show covariance of disease impact across wide-spread regions of the cortex, whereas temporal epicenters show highest regional correlations within temporal, and no correlations with frontal regions (see **Figure S5**).

Distribution of NeuroSynth functional terms along gradient bins

In addition to the 2D space framed by the two transdiagnostic gradients within which cognitive terms were situated, see **Figure 2F** of the main manuscript, we also investigated the position of cognitive terms along each gradient separately. Following the same strategy and using the same 24 NeuroSynth cognitive terms, we binned each gradient into five-percentile bins. Regions of the same bin formed a region of interest (ROI), yielding 20 ROIs for each gradient. These ROIs were then tested for their overlap with meta-analytic ROIs associated with each of the 24 cognitive terms via z-statistics. The magnitude of an average z-value at a ROI (i.e., a position along the gradient) reflects the strength of its association with a certain functional task activation. We sorted the topic terms by their weighted mean position along both gradients, revealing systematic shifts in functional networks along transdiagnostic axes of co-alteration (**Figure S6**). While G1 segregated sensory ('auditory', 'multisensory') from higher order cognitive functions ('Cognitive control', 'inhibition'), G2 distinguished sensory ('auditory', 'multisensory') from perception/attention related functions ('visuospatial', 'attention').

Disorder-specific epicenters and their overlap with transdiagnostic epicenters

In order to investigate to which degree transdiagnostic epicenters are also observed in each disorder, we repeated the epicenter mapping approach using normative HCP connectivity data (rs-fMRI and DTI) in combination with ENIGMA Cohen's *d* maps. For each disorder, we systematically correlated each parcel's normative functional or structural connectivity profile with the absolute Cohen's *d* map and identified parcel's at $p_{spin} < 0.05$ as potential disease epicenters. We then computed the overlap between disorder-specific and transdiagnostic epicenters in percent (See **Figure S7**).

SUPPLEMENTARY FIGURES

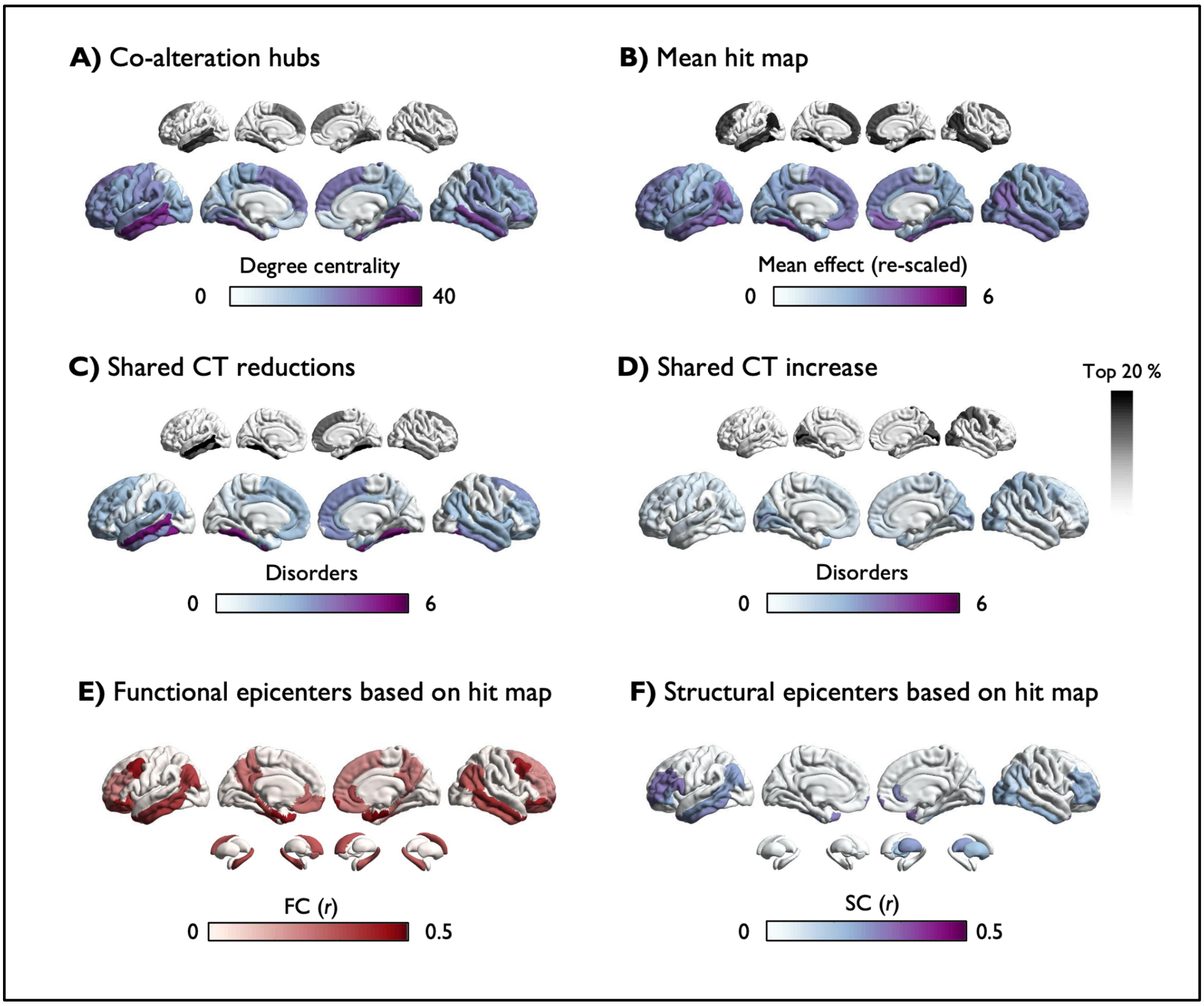

Figure S1. *Correspondence between Co-alteration hubs, shared illness effects, and epicenters.* **A)** Co-alteration hubs. **B)** Hit map based on average absolute Cohen's *d* values, rescaled between 0 and 1 within disorders. Overlaps in cortical thickness (CT) reductions **(C)** and increase **(D)**. In subplots **A - D)**, black and white brain images show a thresholded version (top 20%) of the brain image in the same subplot. **E)** and **F)** depict disease epicenters computed based on the hit map (B) for functional and structural connectivity, respectively.

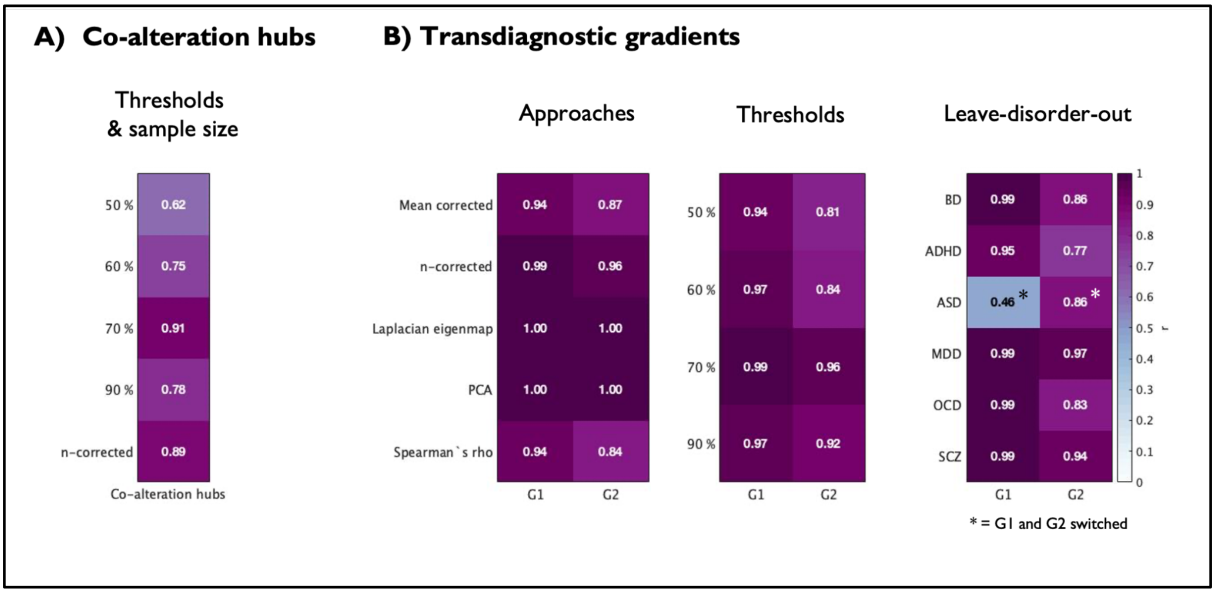

Figure S2. *Robustness of co-alteration hubs and transdiagnostic gradients to parameter manipulations.* Values indicate correlation with original hubs/gradients after parameter manipulation. **A)** Co-alteration hubs based on co-alteration matrix with different cut-offs or corrected for sample-size (n-corrected) per disorder. **B)** Left: Corrected for average illness effects and sample size, or Laplacian eigenmap or principal component analysis (PCA) as dimensionality reduction techniques, or co-alterations based on spearman's rho. Middle: Co-alteration matrix cut-offs. Right: Constructing gradients based on five disorders only, highlighting the contribution of single disorders. *G1 and G2 are switched for autism spectrum disorder (ASD). BD = Bipolar disorder, ADHD = Attention-deficit/hyperactivity disorder, MDD = Major depressive disorder, OCD = Obsessive compulsive disorder, SCZ = Schizophrenia spectrum disorder.

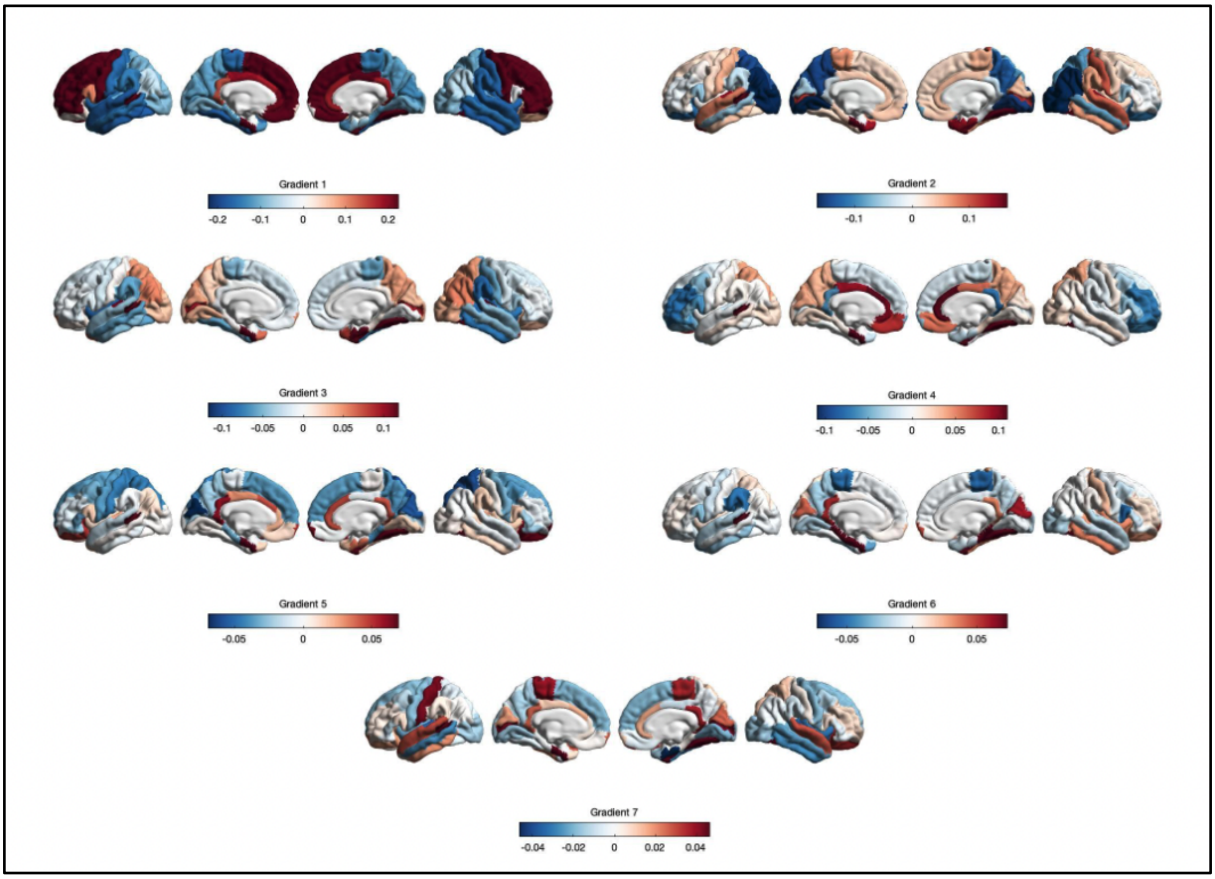

Figure S3. *Overview of all gradients computed from cross-disorder correlation matrix using diffusion embedding.*

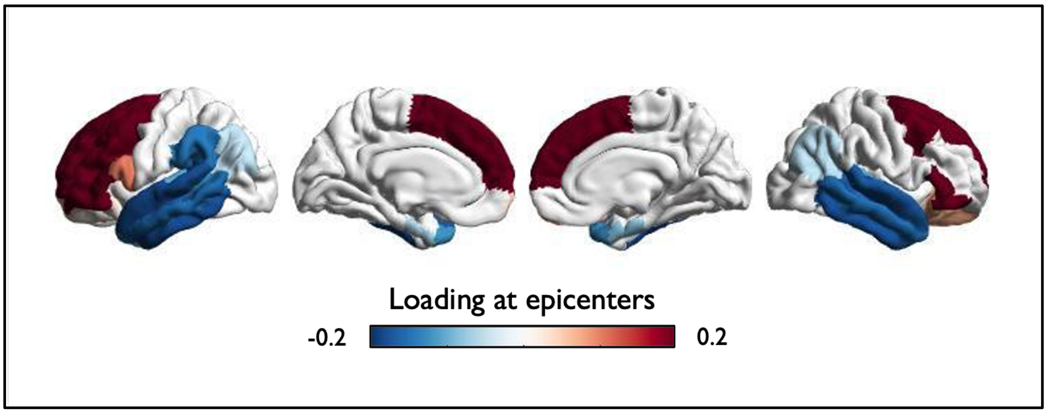

Figure S4. *Gradient loadings at epicenters.* Principal axis (G1) masked by significant functional epicenters, demonstrating that epicenters are strongly placed towards apices of the gradient. Red and blue colors indicate opposite apices of G1.

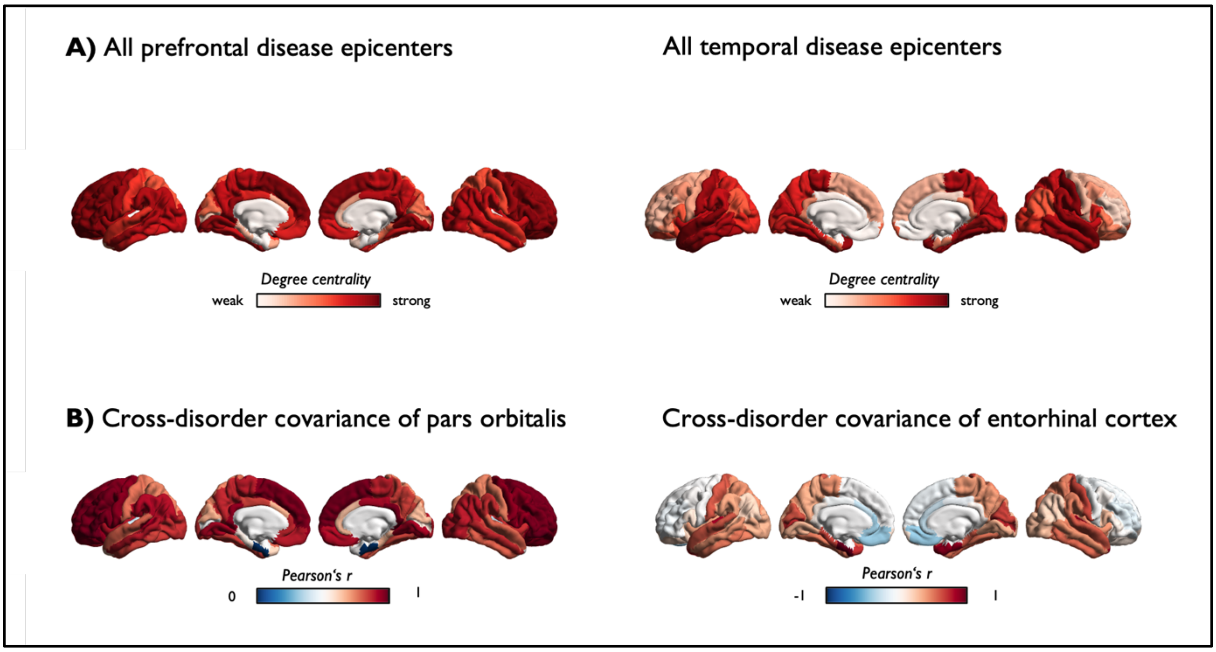

Figure S5. *G1 captures segregation of functional disease epicenters.* **A)** Depicts degree centrality of frontal (left) and temporal (right) functional disease epicenters computed as the sum of whole-brain covariance of cross-disorder disease impact for respective frontal and temporal parcels. **B)** Shows isolated covariance patterns for the two most likely disease epicenters representative for frontal (left pars orbitalis) and temporal (left entorhinal cortex) structures.

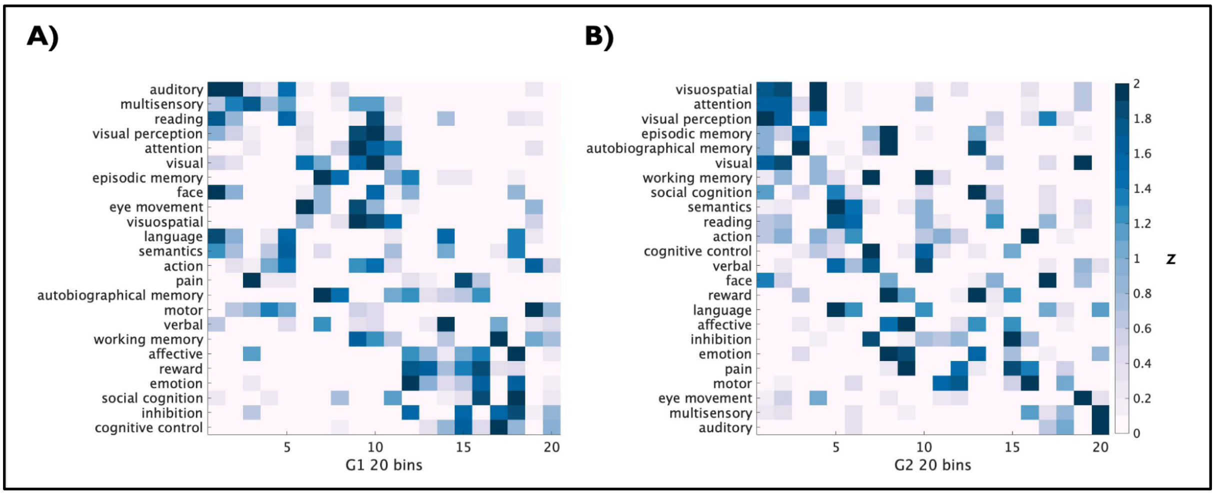

Figure S6. *Meta-analysis for 24 cognitive terms obtained from NeuroSynth*[4] *along the principal (G1) and secondary gradient (G2).* We computed parcel-wise *z*-statistics, capturing node-function associations, and calculated the center of gravity of each function along 20 five-percentile bins of G1 **(A)** and G2 **(B)**. Function terms are ordered by the weighted mean of their location along the gradients.

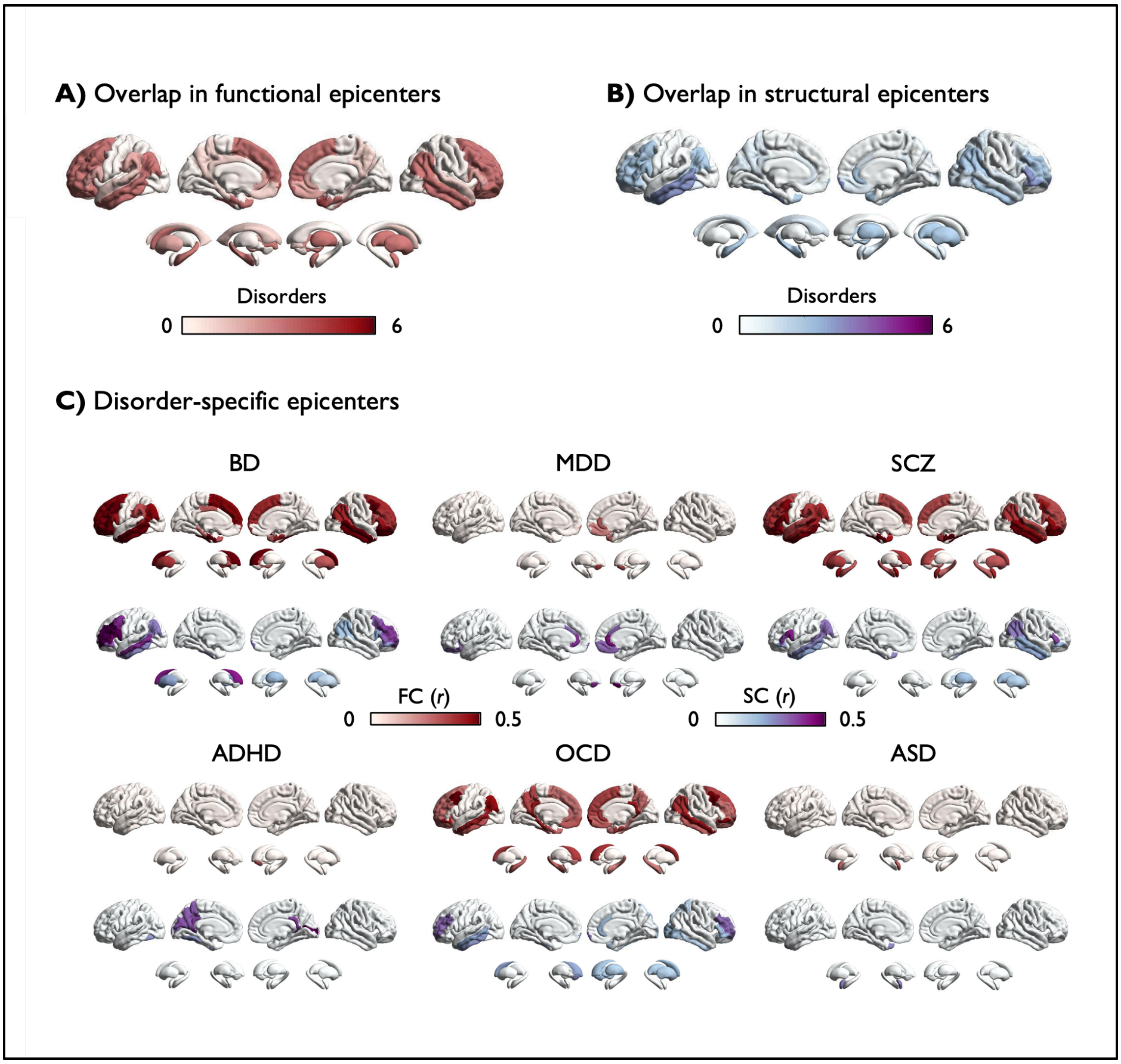

Figure S7. *Overlaps in disorder-specific disease epicenters.* In order to examine to which degree transdiagnostic epicenters also reflect individual disorder's epicenters, we quantified the overlap of functional **(A)** and structural **(B)** epicenters. Epicenter maps for each disorder were binarized, labeling a region as epicenter or no epicenter, and then summed, reflecting in how many disorders a region forms an epicenter. Epicenter maps for individual disorders are depicted in **C)**.

SUPPLEMENTARY TABLES

Table S1. Cohen's *d* values describing case-control differences in cortical thickness for 6 mental disorders used for transdiagnostic analyses.

ROI	ADHD	ASD	BD	MDD	OCD	SCZ
L banks sts	0.01	-0.07	-0.21	-0.06	-0.06	-0.35
L caudal anterior cingulate	-0.11	0.03	-0.10	-0.04	0.00	-0.12
L caudal middle frontal	-0.02	0.06	-0.27	-0.01	-0.09	-0.36
L cuneus	0.11	-0.06	-0.06	0.05	-0.04	-0.20
L entorhinal	-0.06	-0.24	-0.04	-0.04	-0.06	-0.20
L fusiform	-0.01	-0.19	-0.29	-0.12	-0.11	-0.49
L inferior parietal	0.08	-0.05	-0.27	-0.06	-0.14	-0.36
L inferior temporal	0.00	-0.16	-0.25	-0.05	-0.09	-0.45
L isthmus cingulate	0.04	0.05	-0.13	-0.10	-0.07	-0.31
L lateral occipital	0.14	-0.02	-0.16	-0.02	-0.07	-0.33
L lateral orbitofrontal	0.03	0.00	-0.22	-0.05	-0.10	-0.40
L lingual	0.11	-0.02	-0.21	0.01	-0.05	-0.35
L medial orbitofrontal	-0.08	0.08	-0.20	-0.13	-0.08	-0.23
L middle temporal	0.02	-0.12	-0.25	-0.09	-0.09	-0.44
L parahippocampal	0.10	-0.11	-0.02	-0.07	-0.06	-0.28
L paracentral	-0.01	-0.05	-0.14	0.00	-0.01	-0.25
L pars opercularis	-0.01	-0.03	-0.29	-0.06	-0.08	-0.38
L pars orbitalis	-0.01	0.04	-0.25	-0.07	-0.05	-0.32
L pars triangularis	-0.03	0.05	-0.27	-0.05	-0.03	-0.34
L pericalcarine	0.04	-0.01	0.02	0.09	0.01	-0.08
L postcentral	0.05	-0.07	-0.10	0.04	-0.02	-0.26
L posterior cingulate	-0.11	0.05	-0.11	-0.10	-0.07	-0.30
L precentral	-0.06	0.09	-0.21	-0.02	-0.02	-0.34
L precuneus	0.04	-0.08	-0.21	-0.02	-0.10	-0.30
L rostral anterior cingulate	-0.09	0.01	-0.15	-0.13	-0.07	-0.18
L rostral middle frontal	0.03	0.11	-0.28	-0.04	-0.10	-0.36
L superior frontal	-0.04	0.11	-0.23	-0.07	-0.07	-0.43
L superior parietal	0.10	-0.09	-0.16	-0.01	-0.06	-0.21

L superior temporal	0.04	-0.15	-0.21	0.01	-0.01	-0.44
L supramarginal	0.02	-0.09	-0.25	-0.05	-0.05	-0.40
L frontal pole	0.08	0.04	-0.12	-0.01	-0.05	-0.21
L temporal pole	-0.02	-0.14	-0.12	0.01	0.03	-0.25
L transverse temporal	-0.02	-0.24	-0.12	-0.04	0.00	-0.25
L insula	-0.01	-0.09	-0.20	-0.11	-0.07	-0.41
R bankssts	0.01	-0.07	-0.13	-0.07	0.01	-0.36
R caudal anterior cingulate	-0.11	0.03	-0.06	-0.08	-0.04	-0.15
R caudal middle frontal	-0.02	0.06	-0.21	0.01	-0.08	-0.32
R cuneus	0.11	-0.06	-0.03	0.05	-0.03	-0.23
R entorhinal	-0.06	-0.24	-0.08	-0.06	0.01	-0.15
R fusiform	-0.01	-0.19	-0.27	-0.12	-0.09	-0.54
R inferior parietal	0.08	-0.05	-0.26	-0.04	-0.14	-0.35
R inferior temporal	0.00	-0.16	-0.19	-0.12	-0.06	-0.44
R isthmus cingulate	0.04	0.05	-0.18	-0.07	-0.05	-0.31
R lateral occipital	0.14	-0.02	-0.22	0.01	-0.07	-0.34
R lateral orbitofrontal	0.03	0.00	-0.21	-0.12	-0.11	-0.36
R lingual	0.11	-0.02	-0.20	-0.01	-0.04	-0.39
R medial orbitofrontal	-0.08	0.08	-0.23	-0.13	-0.10	-0.24
R middle temporal	0.02	-0.12	-0.22	-0.09	-0.10	-0.38
R parahippocampal	0.10	-0.11	-0.09	-0.06	-0.08	-0.29
R paracentral	-0.01	-0.05	-0.14	-0.01	0.01	-0.22
R pars opercularis	-0.01	-0.03	-0.25	-0.02	-0.06	-0.42
R pars orbitalis	-0.01	0.04	-0.24	-0.07	-0.07	-0.34
R pars triangularis	-0.03	0.05	-0.23	-0.03	-0.06	-0.37
R pericalcarine	0.04	-0.01	0.02	0.08	0.03	-0.09
R postcentral	0.05	-0.07	-0.08	0.03	0.04	-0.28
R posterior cingulate	-0.11	0.05	-0.17	-0.09	-0.06	-0.31
R precentral	-0.06	0.09	-0.18	-0.02	-0.04	-0.32
R precuneus	0.04	-0.08	-0.19	0.01	-0.10	-0.30
R rostral anterior cingulate	-0.09	0.01	-0.09	-0.10	0.01	-0.12
R rostral middle frontal	0.03	0.11	-0.26	-0.04	-0.09	-0.31

R superior frontal	-0.04	0.11	-0.26	-0.08	-0.04	-0.40
R superior parietal	0.10	-0.09	-0.16	0.03	-0.05	-0.22
R superior temporal	0.04	-0.15	-0.19	-0.03	0.01	-0.44
R supramarginal	0.02	-0.09	-0.18	-0.05	0.00	-0.39
R frontal pole	0.08	0.04	-0.10	-0.06	0.02	-0.21
R temporal pole	-0.02	-0.14	-0.06	0.01	0.02	-0.24
R transverse temporal	-0.02	-0.24	-0.11	-0.05	-0.02	-0.26
R insula	-0.01	-0.09	-0.17	-0.12	-0.07	-0.41

Cohen's *d* values were accessed through the ENIGMA Toolbox[3] and for adult samples (except for Autism spectrum disorder (ASD), for which data was only available for a pooled sample including younger subjects). Data was collected and analyzed by respective ENIGMA working groups (Attention-deficit/hyperactivity disorder (ADHD)[7], Autism spectrum disorder (ASD)[8], Bipolar disorder (BD)[9], Major depressive disorder (MDD)[10], Obsessive compulsive disorder (OCD)[11], Schizophrenia (SCZ)[12]) and adjusted for different combinations of age, sex, scan site/scanner differences, intracranial volume, and intelligence quotient effects (see **Table S2**). ROI = Region of interest.

Table S2. Sample Demographics.

Disorder	sites	Mean age (cases / controls)	% female (cases / controls)	IQ (cases / controls)	*n*	Covariates
**Schizophrenia spectrum[12]**	39	32.3[a] / 34.5[a]	34 / 47		Cases: 4474<br>Controls: 5098<br>Total: 9572	age, sex, scan site
**Attention deficit hyperactivity disorder[7]**	36	32.97	25.9 / 40.2[†]		Cases: 733<br>Controls: 539<br>Total: 1272	age, sex, scan site*
**Autism spectrum disorder[8]**	49	15.4 / 15.8	14.3 / 23.8	103 / 111	Cases: 1571<br>Controls: 1651<br>Total: 3222	age, sex, IQ, scan site*
**Bipolar disorder[9]**	28	38.4[a] / 35.6[a]	50.3 / 54.8		Cases: 1837<br>Controls: 2582<br>Total: 4419	age, sex, scan site*
**Major depressive disorder[10]**	20	44.8[a] / 54.6[a]	61.7 / 52.6		Cases: 1911<br>Controls: 7663<br>Total: 9574	age, sex, scan site
**Obsessive-compulsive disorder[11]**	27	32.1 / 30.5	49.5 / 50.3		Cases: 1498<br>Controls: 1436<br>Total: 2934	age, sex, scan site

Adapted from Radonjić et al.[13]. [a] = weighted mean computed by Radonjić et al.[13]. * = In this study, site was included as a random effect in a mixed-effect model and not as a covariate. [†] = Information from the whole consortium sample including all age groups. IQ = Intelligence quotient.

Table S3. 232 Genes for which spatial transcription patterns correlated significantly with the principal transdiagnostic gradient ($P_{spin} < .01$).

Gene symbol	*r*	P_{spin}
PRRX1	0.8	0.002
ZIC1	0.74	0.001
CTXN3	0.71	0.001
RPH3AL	0.7	0.001
CD6	0.69	0.004
LAMA2	0.68	0.003
HSPB8	0.67	0.001
KRT31	0.67	0.001
WNT10A	0.67	0.002
ACTC1	0.67	0.002
YBX2	0.66	0
GORAB	0.65	0.003
FOXF2	0.65	0.009
CCDC80	0.64	0
CD38	0.64	0.005
TCERG1L	0.64	0.001
TEX30	0.64	0.003
NRP1	0.64	0.008
CREB3L3	0.63	0.002
SPRY4	0.63	0.001
CBLN2	0.62	0.004
PCDH10	0.62	0.001
FAM213A	0.62	0.003
SULF1	0.62	0.007
HDAC9	0.62	0.001
ARHGAP25	0.61	0.007
RTP1	0.6	0.004
AEBP1	0.6	0.002
TSPAN33	0.6	0.003
PROCA1	0.6	0.004
TNFRSF14	0.59	0.004
RBP1	0.59	0.001
WFDC1	0.59	0.006
GALNT16	0.59	0.005
AZIN2	0.58	0
MRAP2	0.58	0.006
DBX2	0.58	0.007
ERICH1	0.58	0
OVOL2	0.58	0.003
ZC2HC1A	0.58	0.006
SCRG1	0.58	0.001
ITGA8	0.58	0.005
KCNN2	0.58	0.001
NT5DC2	0.58	0.005
HSPB3	0.57	0.007
CYP51A1	0.57	0.008
TMEM117	0.57	0.007
C6orf62	0.57	0.002
ATP2C2	0.56	0.002
CHRNA6	0.56	0.002
BAIAP2L2	0.55	0.003
TNC	0.55	0.002
RAB3C	0.55	0.007
VIT	0.55	0.005
NECTIN3	0.55	0.001
ZIC3	0.55	0.001
PPEF1	0.55	0.003
COL11A1	0.55	0.007
LINC02217	0.55	0.003
HRH3	0.55	0.007
GPR26	0.55	0
MEIS3P1	0.55	0.003
TMTC3	0.54	0.001
TGFBI	0.54	0.008
CCDC110	0.54	0.008
CMTM4	0.54	0.005
NANOS3	0.54	0.001
ASB6	0.53	0.004
MELTF	0.53	0.001
ASB2	0.53	0.004
FLJ30901	0.53	0.006
TRAF3	0.53	0.008
PLCH1	0.52	0.009
CLIC5	0.52	0.005
ADTRP	0.51	0.008
CASC10	0.51	0.007
BNIP3	0.51	0.006
MGAT4C	0.51	0.009
STBD1	0.5	0.009
PCDHB4	0.5	0.006
CLU	0.5	0.005
CCNYL1	0.5	0.001
PCBP3	0.49	0.009
MPPED1	0.49	0.003
MPP6	0.49	0.007
SCYL3	0.49	0.008
GMPPB	0.49	0.006
GBAP1	0.49	0.008
SC5D	0.49	0.004
GULP1	0.49	0
SMPD1	0.49	0.007
GRM3	0.49	0.007
NMNAT3	0.49	0.005
PNPLA3	0.48	0.008
CAMK1G	0.48	0.006
GOLPH3L	0.48	0.001
ZNF704	0.48	0.007
GKAP1	0.48	0.006
TUBA4A	0.47	0.008
SORCS2	0.47	0.003
TFRC	0.47	0.008
DUSP6	0.47	0.008
CNIH3	0.47	0.003
LRRC4C	0.47	0.008
FREM3	0.47	0.009
ATP7A	0.47	0.009

RNF182	*0.47*	*0.007*
ITGB5	*0.46*	*0.007*
C2orf40	*0.46*	*0.001*
CCDC102B	*0.46*	*0.007*
DACT3	*0.46*	*0.005*
HACD1	*0.46*	*0.009*
COL23A1	*0.46*	*0.006*
PART1	*0.45*	*0.009*
DCUN1D3	*0.45*	*0.004*
SNORC	*0.45*	*0.004*
NXPH2	*0.45*	*0.009*
OSTN	*0.44*	*0.006*
ADCYAP1	*0.44*	*0.004*
DUSP3	*0.44*	*0.006*
RTP4	*0.42*	*0.009*
SLC6A13	*0.42*	*0.005*
PTPA	*0.42*	*0.007*
TAPT1	*0.42*	*0.009*
TUBA1B	*0.42*	*0.003*
TCIM	*0.41*	*0.005*
HCN3	*0.41*	*0.009*
PIK3CD	*0.41*	*0.009*
CNTNAP2	*0.4*	*0.008*
FAM234B	*0.4*	*0.003*
RRP8	*0.4*	*0.008*
OSCAR	*0.4*	*0.009*
KAT5	*0.4*	*0.006*
LOC108783654	*0.4*	*0.009*
SLC25A42	*0.4*	*0.009*
HDAC5	*0.39*	*0.006*
TRMT61A	*0.38*	*0.006*
NRSN1	*0.37*	*0.004*
GPR22	*0.37*	*0.008*
LRRC32	*0.37*	*0.003*
TRABD2A	*0.37*	*0.009*
PLK3	*0.37*	*0.008*
PRKX	*0.36*	*0.009*
KLF11	*0.35*	*0.007*
GNLY	*0.35*	*0.009*
TMEM232	*0.34*	*0.007*
PEX16	*-0.29*	*0.006*
FAM163B	*-0.3*	*0.008*
ZFYVE27	*-0.31*	*0.009*
IGFBPL1	*-0.33*	*0.009*
ALPK1	*-0.34*	*0.005*
LINC00167	*-0.34*	*0.004*
ANAPC13	*-0.35*	*0.004*
DUSP11	*-0.36*	*0.008*
VAMP5	*-0.37*	*0.009*
ZBTB49	*-0.37*	*0.003*
TBC1D17	*-0.38*	*0.005*
FAM118B	*-0.38*	*0.005*
MPPE1	*-0.38*	*0.009*
BCL2L11	*-0.39*	*0.008*
MAML3	*-0.4*	*0.002*
NELFA	*-0.4*	*0.009*
EXOSC2	*-0.4*	*0.005*
ZNF766	*-0.4*	*0.009*

ZNF594	*-0.41*	*0.006*
SRSF11	*-0.41*	*0.009*
PKP2	*-0.41*	*0.009*
SRSF4	*-0.41*	*0.004*
AP1G2	*-0.41*	*0.007*
CCDC59	*-0.42*	*0.008*
ZNF781	*-0.42*	*0.008*
DDI2	*-0.43*	*0.008*
CXorf56	*-0.44*	*0.007*
HAUS4	*-0.44*	*0.004*
OLFML2A	*-0.44*	*0.009*
GPR83	*-0.45*	*0.001*
EPCAM	*-0.45*	*0.007*
SPEF1	*-0.45*	*0.005*
RNASEH1	*-0.45*	*0.005*
LDB1	*-0.46*	*0.007*
AGAP11	*-0.46*	*0.005*
LOC100130950	*-0.46*	*0.009*
TMEM19	*-0.47*	*0.001*
LOC100131289	*-0.47*	*0.008*
MRPL50	*-0.47*	*0.003*
MINPP1	*-0.47*	*0.003*
GPHN	*-0.48*	*0.006*
CRABP2	*-0.48*	*0.003*
LONRF3	*-0.48*	*0.007*
ALKBH5	*-0.48*	*0.007*
ACKR1	*-0.49*	*0.006*
STRIP2	*-0.49*	*0.002*
ITFG2	*-0.49*	*0.007*
WNT2B	*-0.49*	*0.002*
FBXO3	*-0.49*	*0.007*
ZFP37	*-0.5*	*0.002*
FGD1	*-0.5*	*0.003*
HEATR3	*-0.5*	*0.007*
GPR19	*-0.5*	*0.007*
TAP2	*-0.5*	*0.003*
HSD17B11	*-0.5*	*0.008*
BTBD3	*-0.51*	*0.001*
KRTCAP3	*-0.51*	*0.006*
PAM	*-0.52*	*0.008*
VCX	*-0.52*	*0.007*
ANKRD20A11P	*-0.52*	*0.005*
PATJ	*-0.53*	*0.001*
MTHFD2L	*-0.54*	*0.007*
FBXO11	*-0.54*	*0.009*
PLOD2	*-0.54*	*0.005*
RIPK1	*-0.54*	*0.009*
DPP6	*-0.54*	*0.008*
WNT3	*-0.55*	*0.008*
GREB1L	*-0.55*	*0.005*
SDHAF4	*-0.56*	*0.008*
MST1R	*-0.56*	*0.008*
PDGFD	*-0.56*	*0.005*
TENM4	*-0.57*	*0.006*
THSD7A	*-0.57*	*0.007*
SLFN11	*-0.58*	*0.005*
LAMP5	*-0.58*	*0.002*
ZNF662	*-0.58*	*0.002*

TPTE2P1	*-0.59*	*0.005*
CDH12	*-0.61*	*0.007*
DSP	*-0.62*	*0.004*
SLC17A6	*-0.66*	*0.006*
CPLX2	*-0.67*	*0.001*

ANKRD20A5P	*-0.69*	*0.002*
C15orf59	*-0.69*	*0.003*
COL27A1	*-0.71*	*0.002*
WDR97	*-0.71*	*0.003*
LXN	*-0.73*	*0*

Table S4. Link between principal (G1) and secondary (G2) transdiagnostic axes of pathological covariance and disease-specific Cohen's *d* maps.

	**ADHD**	**ASD**	**BD**	**MDD**	**SCZ**	**OCD**
**G1**	r = -0.52, p_{spin} = 0.002	r = 0.82, p_{spin} = 0.001	r = -0.28, p_{spin} = 0.075	r = -0.29, p_{spin} = 0.079	r = 0.11, p_{spin} = 0.33	r = -0.20, p_{spin} = 0.021
**G2**	r = -0.58, p_{spin} < .0001	r = -0.26, p_{spin} = 0.050	r = 0.42, p_{spin} = 0.001	r = 0.001, p_{spin} = 0.501	r = 0.24, p_{spin} = 0.041	r = 0.6, p_{spin} = 0.001

Supplementary References

1. Van Essen, D. C. *et al.* The Human Connectome Project: A data acquisition perspective. *Neuroimage* **62**, 2222–2231 (2012).
2. Desikan, R. S. *et al.* An automated labeling system for subdividing the human cerebral cortex on MRI scans into gyral based regions of interest. *Neuroimage* **31**, 968–980 (2006).
3. Larivière, S. *et al.* The ENIGMA Toolbox: multiscale neural contextualization of multisite neuroimaging datasets. *Nat. Methods* **18**, 698–700 (2021).
4. Alexander-Bloch, A. F. *et al.* On testing for spatial correspondence between maps of human brain structure and function. *Neuroimage* **178**, 540–551 (2018).
5. Vos de Wael, R. *et al.* BrainSpace: a toolbox for the analysis of macroscale gradients in neuroimaging and connectomics datasets. *Commun Biol* **3**, 103 (2020).
6. Yarkoni, T., Poldrack, R. A., Nichols, T. E., Van Essen, D. C. & Wager, T. D. Large-scale automated synthesis of human functional neuroimaging data. *Nat. Methods* **8**, 665–670 (2011).
7. Hoogman, M. *et al.* Brain Imaging of the Cortex in ADHD: A Coordinated Analysis of Large-Scale Clinical and Population-Based Samples. *Am J Psychiatry* **176**, 531–542 (2019).
8. van Rooij, D. *et al.* Cortical and Subcortical Brain Morphometry Differences Between Patients With Autism Spectrum Disorder and Healthy Individuals Across the Lifespan: Results From the ENIGMA ASD Working Group. *Am J Psychiatry* **175**, 359–369 (2018).
9. Hibar, D. P. *et al.* Cortical abnormalities in bipolar disorder: an MRI analysis of 6503 individuals from the ENIGMA Bipolar Disorder Working Group. *Mol Psychiatry* **23**, 932–942 (2018).
10. Schmaal, L. *et al.* Cortical abnormalities in adults and adolescents with major depression based on brain scans from 20 cohorts worldwide in the ENIGMA Major Depressive Disorder Working Group. *Mol Psychiatry* **22**, 900–909 (2017).
11. Boedhoe, P. S. W. *et al.* Cortical Abnormalities Associated With Pediatric and Adult Obsessive-Compulsive Disorder: Findings From the ENIGMA Obsessive-Compulsive Disorder Working Group. *Am J Psychiatry* **175**, 453–462 (2018).
12. van Erp, T. G. M. *et al.* Cortical Brain Abnormalities in 4474 Individuals With Schizophrenia and 5098 Control Subjects via the Enhancing Neuro Imaging Genetics Through Meta Analysis (ENIGMA) Consortium. *Biol Psychiatry* **84**, 644–654 (2018).
13. Radonjic, N. V. *et al.* Structural brain imaging studies offer clues about the effects of the shared genetic etiology among neuropsychiatric disorders. *Mol Psychiatry* (2021) doi:10.1038/s41380-020-01002-z.
