# Supplementary material for: Coordinated cortical thickness alterations across six neurodevelopmental and psychiatric disorders
Source: Nat Commun. 2022 Nov 11;13:6851. doi: 10.1038/s41467-022-34367-6 (PMC9652311; doi:10.1038/s41467-022-34367-6)
Supplement: Supplementary file 3 — Reporting Summary [file 41467_2022_34367_MOESM3_ESM.pdf]

nature portfolio

Corresponding author(s): Meike D Hettwer & Sofie L Valk

Last updated by author(s): 11.10.2022

Reporting Summary

Nature Portfolio wishes to improve the reproducibility of the work that we publish. This form provides structure for consistency and transparency in reporting. For further information on Nature Portfolio policies, see our Editorial Policies and the Editorial Policy Checklist.

Statistics

For all statistical analyses, confirm that the following items are present in the figure legend, table legend, main text, or Methods section.

n/a	Confirmed	
☐	☒	The exact sample size (n) for each experimental group/condition, given as a discrete number and unit of measurement
☒	☐	A statement on whether measurements were taken from distinct samples or whether the same sample was measured repeatedly
☐	☒	The statistical test(s) used AND whether they are one- or two-sided<br>*Only common tests should be described solely by name; describe more complex techniques in the Methods section.*
☐	☒	A description of all covariates tested
☐	☒	A description of any assumptions or corrections, such as tests of normality and adjustment for multiple comparisons
☐	☒	A full description of the statistical parameters including central tendency (e.g. means) or other basic estimates (e.g. regression coefficient) AND variation (e.g. standard deviation) or associated estimates of uncertainty (e.g. confidence intervals)
☐	☒	For null hypothesis testing, the test statistic (e.g. F, t, r) with confidence intervals, effect sizes, degrees of freedom and P value noted<br>*Give P values as exact values whenever suitable.*
☒	☐	For Bayesian analysis, information on the choice of priors and Markov chain Monte Carlo settings
☒	☐	For hierarchical and complex designs, identification of the appropriate level for tests and full reporting of outcomes
☐	☒	Estimates of effect sizes (e.g. Cohen's d, Pearson's r), indicating how they were calculated

Our web collection on statistics for biologists contains articles on many of the points above.

Software and code

Policy information about availability of computer code

Data collection	We used the ENIGMA Toolbox (v.1.1.3; https://enigma-toolbox.readthedocs.io/en/latest/) to access ENIGMA disease maps, normative connectivity data from Human Connectome Project Data, and transcriptomic maps from the Allen human brain atlas.
Data analysis	Custom code generated for this project was made publicly available under https://github.com/CNG-LAB/cngopen/tree/main/transdiagnostic_gradients. Our analysis code makes use of open software: Gradient mapping analyses were carried out using BrainSpace (v. 0.1.2; https://brainspace.readthedocs.io/en/latest/) and epicenters were computed using code from the ENIGMA Toolbox (v. 1.1.3; https://enigma-toolbox.readthedocs.io/en/latest/). Visualizations were carried out using BrainStat (v. 0.3.6; https://github.com/MICA-MNI/BrainStat) in combination with ColorBrewer (v. 1.0.0; https://github.com/scottclowe/cbrewer2). Genetic analyses were performed using the GAMBA Toolbox (2021; https://github.com/dutchconnectomelab/GAMBA-MATLAB) and the cell-specific enrichment analysis tool (v. 1.1; http://genetics.wustl.edu/jdlab/csea-tool-2/).

For manuscripts utilizing custom algorithms or software that are central to the research but not yet described in published literature, software must be made available to editors and reviewers. We strongly encourage code deposition in a community repository (e.g. GitHub). See the Nature Portfolio guidelines for submitting code & software for further information.

March 2021

Data

Policy information about availability of data

All manuscripts must include a data availability statement. This statement should provide the following information, where applicable:

- Accession codes, unique identifiers, or web links for publicly available datasets
- A description of any restrictions on data availability
- For clinical datasets or third party data, please ensure that the statement adheres to our policy

All data analyzed in this manuscript were obtained from open-access sources. Disorder-specific Cohen's d maps derived from ENIGMA meta-analyses were accessed via the ENIGMA Toolbox (v. 1.1.3; https://enigma-toolbox.readthedocs.io/en/latest/; Lariviere et al, 2021). Through the toolbox, we also accessed normative connectivity data from a Human Connectome Project young adult sample (HCP; http://www.humanconnectome.org/; Van Essen, 2012), the von Economo-Koskinas cytoarchitectonic atlas (Triarhou, 2007), and gene transcriptomic data from the Allen human brain atlas (https://human.brain-map.org/). The functional meta-analysis was based on the NeuroSynth database (https://neurosynth.org/). Developmental enrichment analyses were based on the Brainspan dataset (https://www.brainspan.org/static/download.html). Data generated for this study were made publicly available under https://github.com/CNG-LAB/cngopen/tree/main/transdiagnostic_gradients. Raw imaging data supporting our findings are not publicly available as they contain information that could compromise the privacy of study participants. There are data sharing restrictions imposed by (i) ethical review boards of the participating sites, and consent documents; (ii) national and trans-national data sharing law, such as GDPR; and (iii) institutional processes, some of which require a signed MTA for limited and predefined data use. However, we welcome sharing data with researchers, requiring only that they submit an analysis plan for a secondary project to the leading team of the Working Group (http://enigma.ini.usc.edu). Once this analysis plan is approved, access to the relevant data will be provided contingent on data availability and local PI approval and compliance with all supervening regulations. If applicable, distribution of analysis protocols to sites will be facilitated. Source data are provided with this paper. Source data are provided with this paper (Supplementary Material).

Field-specific reporting

Please select the one below that is the best fit for your research. If you are not sure, read the appropriate sections before making your selection.

☒ Life sciences ☐ Behavioural & social sciences ☐ Ecological, evolutionary & environmental sciences

For a reference copy of the document with all sections, see nature.com/documents/nr-reporting-summary-flat.pdf

Life sciences study design

All studies must disclose on these points even when the disclosure is negative.

Sample size	We studied summary statistics from 12,024 patients and 18,969 controls from the Enhancing NeuroImaging Genetics (ENIGMA) consortium. The sample sizes of ENIGMA data are among the largest clinical samples collected to date and summary statistics are based on the maximum number of participants who met inclusion and data quality criteria. Our work is based on these summary statistics which did not allow us to influence the sample size.
Data exclusions	Exclusion of single participants due to MRI contra-indications, scan quality, co-morbidities etc. was performed by individual sites contributing to the ENIGMA consortium. Here, we used summary statistics which did not allow us to additionally exclude further single subjects. Wherever possible we restricted our analyses to adult samples to avoid biases related to developmental effects/age differences.
Replication	There is no open dataset of comparable size and range of disorders that could have been used for a replication.
Randomization	Our study did not include a random allocation of participants to groups, as groups were defined by clinical diagnoses. However, we performed spin tests (i.e. a spatial permutation controlling for auto-correlations), which randomly rotate cortical maps 1000 times for spatial association analyses.
Blinding	N/A Due to the use of summary statistics, investigators conducting the analyses did not have access to single subject information. Diagnostic labels of group statistics were not masked during analyses. However, as the study aimed to identify shared/transdiagnostic features rather than differences between disorders/samples, knowledge on disorder labels could not impact the analyses or results.

Reporting for specific materials, systems and methods

We require information from authors about some types of materials, experimental systems and methods used in many studies. Here, indicate whether each material, system or method listed is relevant to your study. If you are not sure if a list item applies to your research, read the appropriate section before selecting a response.

Materials & experimental systems

n/a	Involved in the study
☒	☐ Antibodies
☒	☐ Eukaryotic cell lines
☒	☐ Palaeontology and archaeology
☒	☐ Animals and other organisms
☐	☒ Human research participants
☒	☐ Clinical data
☒	☐ Dual use research of concern

Methods

n/a	Involved in the study
☒	☐ ChIP-seq
☒	☐ Flow cytometry
☐	☒ MRI-based neuroimaging

Human research participants

Policy information about studies involving human research participants

Population characteristics	Schizophrenia sample: 39 sites, mean age: 32.3 (cases) / 34.5 (controls), % female: 34 (cases) / 47 (controls), N = 9572 (4474 cases, 5098 controls);<br>Autism spectrum disorder: sample: 49 sites, mean age: 15.4 (cases) / 15.8 (controls), % female: (14.3 (cases) / 23.8 (controls), IQ: 103 (cases) /111 (controls) N = 3222 (1571 cases, 1651 controls);<br>ADHD: 36 sites, mean age: 32.97, % female: (25.9 (cases) / 40.2 (controls), N =1272 (733 cases, 539 controls);<br>Bipolar disorder: 28 sites, mean age: 38.4 (cases) / 35.6 (controls), % female: 38.4 (cases) / 35.6 (controls), N = 4419 (1837 cases, 2582 controls);<br>Major depressive disorder: 20 sites, mean age: 44.8 (cases), 54.6 (controls), % female: 61.7 (cases) / 52.6 (controls), N = 9574 (1911 cases, 7663 controls);<br>Obsessive-compulsive disorder: 27 sites, mean age: 32.1 (cases) / 30.5 (controls), % female: 32.1 (cases) / 30.5 (controls), N = 2934 (1,498 cases, 1,436 controls);
Recruitment	Patients and healthy controls were recruited in a collaborative effort through individual ENIGMA sites across the whole world.
Ethics oversight	The ENIGMA data collections were performed in accordance with local Institutional Review Board guidelines. In accordance with HIPAA guidelines, all used datasets were fully anonymized, with no protected health information included. In general, used summary statistic did not include any information on individual subjects.

Note that full information on the approval of the study protocol must also be provided in the manuscript.

Magnetic resonance imaging

Experimental design

Design type	structural MRI (cortical thickness)
Design specifications	N/A
Behavioral performance measures	N/A

Acquisition

Imaging type(s)	structural /cortical thickness
Field strength	1.5-3T depending on acquisition site. This information is included in the original ENIGMA publications referenced in the main manuscript.
Sequence & imaging parameters	Imaging parameters slightly differ depending on acquisition site. This information is included in the original ENIGMA publications referenced in the main manuscript.
Area of acquisition	whole brain

Diffusion MRI ☐ Used ☒ Not used

Preprocessing

Preprocessing software	Freesurfer standart recon-all pipieline. Standart ENIGMA workflows such as Quality Control are documented in detail under: http://enigma.ini.usc.edu/protocols/imaging-protocols/
Normalization	recon-all default FreeSurfer pipeline
Normalization template	Structural data was normalized to the fsaverage5 template. Subcortex was registered to MNI space.

Noise and artifact removal

Segmentation errors and other artifacts were checked by individual ENIGMA sites according to standart ENIGMA quality control (including parcel-wise outlier detection and manual/visual checks) - as described here: http://enigma.ini.usc.edu/protocols/imaging-protocols/

Volume censoring

n/a

Statistical modeling & inference

Model type and settings

We computed a cross-disorder covariance matrix of illness effects (i.e. inter-regional correlations of case-control differences across included disorders). Based on this matrix, we defined a "co-alteration hub map" via degree centrality (i.e. the sum of strong connections) which we correlated with seed-based connectivity profiles to identify disease epicenters (corrected for spatial auto-correlation via spin tests/1000 permutations). Moreover, we derived cortex-wide transdiagnostic gradients capturing maximally different co-alteration patterns via manifold learning (diffusion embedding).

Effect(s) tested

n/a

Specify type of analysis: ☒ Whole brain ☐ ROI-based ☐ Both

Statistic type for inference
(See Eklund et al. 2016)

We performed spin tests (1000 permutations) where applicable (when comparing two cortical maps)

Correction

Permutations (spin tests for phenotypic maps, null-coexpression and null-brain models for transcriptomic maps)

Models & analysis

n/a	Involved in the study
☐	☒ Functional and/or effective connectivity
☐	☒ Graph analysis
☒	☐ Multivariate modeling or predictive analysis

Functional and/or effective connectivity

Pearson Correlation & Fisher's z-transform

Graph analysis

We computed hubs via degree centrality both for HCP connectivity data and for a structural covariance network. Hubs were based on binarized graphs at the group level and identified as regions with a high sum of connections (thresholded at 80%; analogous to degree centrality).
